# Marine Bacterial Secondary Metabolites: A Treasure House for Structurally Unique and Effective Antimicrobial Compounds

**DOI:** 10.3390/md19100530

**Published:** 2021-09-23

**Authors:** Ramanathan Srinivasan, Arunachalam Kannappan, Chunlei Shi, Xiangmin Lin

**Affiliations:** 1 Fujian Provincial Key Laboratory of Agroecological Processing and Safety Monitoring, School of Life Sciences, Fujian Agriculture and Forestry University, Fuzhou 350002, China; 2 Key Laboratory of Crop Ecology and Molecular Physiology, Fujian Agriculture and Forestry University, Fuzhou 350002, China; 3 State Key Laboratory of Microbial Metabolism, MOST-USDA Joint Research Center for Food Safety, School of Agriculture and Biology, Shanghai Jiao Tong University, Shanghai 200240, China; kannanacmdu@sjtu.edu.cn (A.K.); clshi@sjtu.edu.cn (C.S.); 4 Key Laboratory of Marine Biotechnology of Fujian Province, Institute of Oceanology, Fujian Agriculture and Forestry University, Fuzhou 350002, China

**Keywords:** antibacterial, antifungal, antimicrobial, antiviral, marine bacteria, marine fauna, marine flora, marine sediments, marine water, secondary metabolites

## Abstract

The prevalence of antimicrobial resistance reduces the effectiveness of antimicrobial drugs in preventing and treating infectious diseases caused by pathogenic organisms, such as bacteria, fungi, and viruses. Because of the burgeoning growth of microbes with antimicrobial-resistant traits, there is a dire need to identify and develop novel and effective antimicrobial agents to treat infections from antimicrobial-resistant strains. The marine environment is rich in ecological biodiversity and can be regarded as an untapped resource for prospecting novel bioactive compounds. Therefore, exploring the marine environment for antimicrobial agents plays a significant role in drug development and biomedical research. Several earlier scientific investigations have proven that bacterial diversity in the marine environment represents an emerging source of structurally unique and novel antimicrobial agents. There are several reports on marine bacterial secondary metabolites, and many are pharmacologically significant and have enormous promise for developing effective antimicrobial drugs to combat microbial infections in drug-resistant pathogens. In this review, we attempt to summarize published articles from the last twenty-five years (1996–2020) on antimicrobial secondary metabolites from marine bacteria evolved in marine environments, such as marine sediment, water, fauna, and flora.

## 1. Introduction

Bacteria are ubiquitous, and they are vital for the preservation of the ecosystem in which we live. Only a small percentage of bacteria are pathogenic. However, the infections caused by pathogenic bacteria significantly affect public health [1,2]. Bacterial infections are often more superficially controlled than fungal and viral infections because of the presence of an antibacterial drugs arsenal with growth inhibitory activity. However, the rapid development of bacterial resistance to antimicrobials is a pressing issue that surpasses the devastating effects of fungal and viral infections [1,3]. The phenomenon of resistance is not equivalently spread across all bacterial species. However, the Infectious Disease Society of America (IDSA) has recognized six bacterial species as highly dangerous owing to their possible multidrug resistance mechanisms and pathogenicity [4]. *Enterococcus faecium*, *Staphylococcus aureus*, *Klebsiella pneumoniae*, *Acinetobacter baumannii*, *Pseudomonas aeruginosa*, and *Enterobacter* sp. are referred to as ESKAPE pathogens. These ESKAPE pathogens are the frequent causes of severe or life-threatening infections, particularly in immunocompromised individuals, critically sick individuals, and children [5,6,7].

Fungal pathogens, such as *Candida* sp. and *Aspergillus* sp., may cause life-threatening infections that lead to illness, and possibly death [8]. For example, *Candida albicans*, a frequent colonizer of gut mucosal surfaces, are among the top five infectious agents responsible for sepsis [9]. Furthermore, invasive candidiasis is the most prevalent fungal infection among hospitalized patients, with a mortality rate of 40% [10]. Similarly, *Aspergillus fumigates* has emerged as a key pathogen in cancer patients. When healthy people inhale conidia of *A. fumigates*, the host’s innate immunity is adequate to eliminate the organism without forming antibody or cell-mediated acquired immunity [11]. On the other hand, *A. fumigates* in immunocompromised individuals increases the risk of developing severe invasive illnesses with mortality rates ranging from 40 to 90% [12].

Viruses are very contagious and cause infections in humans. Due to horizontal transmission, we have seen many viral pandemics. Such pandemics have culminated in health and economic crises. Since December 2019, the entire world has been in the grips of another pandemic viral outbreak. This time, it is severe acute respiratory syndrome coronavirus 2 that is the causative agent of the pandemic. Since then, the viral pandemic has claimed millions of lives and wreaked havoc on the global economy [13,14]. Additionally, humankind is currently still battling a past pandemic caused by the human immunodeficiency virus. The acquired immunodeficiency syndrome (HIV/AIDS) outbreak has infected 37 million individuals globally [15].

The arsenal of chemical compounds used to treat human microbial infections was considered adequate after discovering most of the antimicrobial classes from the 1940s to 1960s, the so-called “Golden Age” of antimicrobial research [16]. However, the rapid development of antimicrobial resistance in microorganisms quickly contradicted the premise of antibiotics. Antimicrobials exert selection pressure on microorganisms, resulting in random chromosomal changes that make the microbes more amenable to living in the environment with antimicrobials, disinfectants, and chemical compounds [17,18,19,20]. The genetic material that encodes for such amenability is quickly replicated and disseminated [21]. In addition, the excessive and inappropriate usage of antimicrobial agents also drastically accelerates the development of drug resistance [22,23]. As a result, pathogenic microorganisms have emerged and reached epidemic and pandemic levels. Antimicrobial-resistant pathogenic microorganisms are classified as a threat to human health by the World Health Organization (WHO), and the Centers for Disease Control and Prevention (CDC) [24,25]. It is also estimated that antimicrobial resistance could cause 10 million deaths per year by 2050, resulting in a global economic loss of up to $100 trillion [26].

Over the past two decades, the number of new antimicrobials on the market has decreased. However, harmful microorganisms have developed a high resistance rate, implying that existing antimicrobials are no longer effective [27,28,29]. As a result, increased efforts toward developing and commercializing new antimicrobial agents are urgently needed [30]. While considerable progress has been achieved in the chemical synthesis and engineering biosynthesis of antimicrobial agents, nature remains the richest and most versatile source for discovering novel antimicrobial agents [31]. To find effective compounds with unique antimicrobial characteristics, researchers from all around the globe are searching meticulously for novel sources in a variety of natural environments. After pointing out the fundamental health risks faced by humankind due to pathogenic organisms, this review explores the contribution of marine bacterial-derived natural products in the fight against microbial infections.

## 2. The Marine Environment: A Goldmine for Natural Product Research

Oceans encompass 70% of the Earth’s surface, and their phylogenetic diversity is considerably greater than that of the terrestrial environment because of the differences in location, temperature, and salinity [32,33]. The ocean is home to an estimated 50–80% of all life on Earth [34]. The oceans are a unique and abundant supply of bioactive compounds for the pharmaceutical industries owing to the enormous genetic and ecological diversity of marine entities [35]. Over the last 50 years, more than 20,000 natural compounds have been identified in the marine environment [36]. This complex ecosystem has been identified in various marine entities, including marine plants, animals, their associated microbes, and the microorganisms derived from marine sediment and marine water [9,37,38,39,40,41]. Most conventional secondary metabolites have been discovered in terrestrial flora and fauna and their associated microorganisms [42,43,44,45,46,47]. These secondary metabolites have previously been found and reported to have effective therapeutic and antimicrobial capabilities [48,49,50,51,52]. However, the rediscovery of known chemical compounds has hampered novel antimicrobials in the terrestrial environment for the treatment of microbial infections [53]. Recently, significant attention has been directed toward marine metabolites, which may provide the key to developing effective therapeutic agents against microbial pathogens, while substantial research on new antimicrobials has been conducted in the terrestrial environment [54]. The natural products obtained from marine environments have various structural characteristics and vary from those found in terrestrial environments [55].

Marine organisms have provided essential components that have shown their potential for use in industrial development as agrochemicals, as well as in cosmetics, fine chemicals, nutritional supplements, and as therapeutic agents for treating several illnesses [56]. Over the last 25 years, the discipline of marine drug discovery has expanded with over 35,000 research articles on natural chemical compounds derived from marine origins [57]. This is also shown by the rapid discovery of new secondary metabolites and a large number of marine-derived therapeutic candidates in clinical trials or awaiting approval [58,59]. Thus, the marine environment is expected to provide the pharmaceutical sector with the next generation of antimicrobial agents [60,61].

## 3. Secondary Metabolites from Marine Bacteria: A Treasure House of Natural Bioactives

Microorganisms are more suited to the large-scale synthesis of bioactive compounds, as they overcome the difficulties associated with acquiring drugs via massive harvesting [62]. Secondary metabolites produced by microorganisms, such as bacteria and fungi, may have effective bioactivity against other microorganisms or the specific physiological conditions of a diseased body. Since the first antibiotic penicillin discovery in the 1920s, it has been assumed that secondary metabolites from microorganisms constitute the primary source of novel secondary metabolites with antimicrobial activities [63]. Microorganisms, particularly bacteria, are considered a fascinating source of structurally diverse and effective bioactive compounds [54,64]. Bacteria from marine water, sediment, and marine organisms can produce a wide range of novel therapeutic compounds with a broad range of applications [65]. The associated marine bacteria use nutrients (e.g., carbon) generated by their host to defend themselves against harmful entities in the environment by secreting active biological chemicals [66]. Furthermore, this selection revealed that, compared to other surface-dwelling bacteria, associated bacterial communities might produce novel antimicrobials to maintain their host position. As a result, it has been shown that these bacteria generate various bioactive compounds [23]. Many bioactive compounds were identified from marine bacteria, such as antibacterial, antiviral, antifungal, antiquorum-sensing, and anticancer substances [67,68,69]. Earlier evidence suggests that bacteria associated with marine organisms generate secondary metabolites attributed to the hosts [65]. The production of such secondary metabolites by marine bacteria opens up new avenues for developing novel natural compounds. Furthermore, marine bacteria are proving to be an exciting source for the development of novel therapeutic agents. For instance, if adequately screened and scrutinized, marine bacteria might provide us with the antimicrobials necessary to fight drug-resistant pathogens for the next 100 years [30].

## 4. Antimicrobial Production by Different Phyla of Marine Bacteria

**Figure 1:**
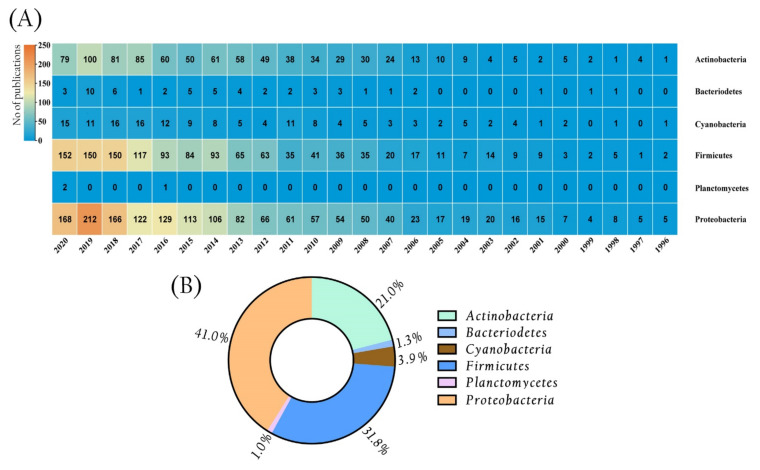
Antimicrobial activity was reported from the main marine bacterial phyla. (**A**) Categorization of articles published between the years 1996–2020, according to bacterial phylum, using the keywords “marine phylum” [‘marine Actinobacteria’, ‘marine Bacteriodetes’, ‘marine Cyanobacteria’, ‘marine Firmicutes’, ‘marine Planctomycetes’, and ‘marine Proteobacteria’], and “antimicrobial activity”. Source: PubMed database (https://pubmed.ncbi.nlm.nih.gov/, accessed on 25 July 2021). (**B**) The pie chart shows the percentage of scholarly articles related to major marine bacterial phyla that have been published for their antimicrobial potentials in the last twenty-five-year study period.

Marine microbes produce about 23,000 bioactive secondary metabolites, according to estimates. Among the different marine microorganisms, the secondary metabolites produced by marine bacteria have many biological activities, including antimicrobial potential [70]. The six phyla of marine bacteria (Actinobacteria, Bacteroidetes, Cyanobacteria, Firmicutes, Planctomycetes, and Proteobacteria) seem to be the frequent primary sources of antimicrobial compounds among the various phyla investigated so far [71,72,73,74,75]. Our literature survey shows the presence of around 834, 53, 148, 1214, 3, and 1565 scholarly articles published between the years 1996–2020 related to antimicrobial activity in the PubMed database with the keywords: “marine Actinobacteria”, “marine Bacteroidetes”, “marine Cyanobacteria”, “marine Firmicutes”, “marine Planctomycetes”, and “marine Proteobacteria”, respectively (Figure 1A). The main phyla linked with antimicrobial activity are Actinobacteria, Firmicutes, and Proteobacteria, which account for more than 93% of the scholarly articles addressed in the last twenty-five years (Figure 1B).

### 4.1. Antimicrobial Potential of Marine Actinobacteria

The phylum Actinobacteria is Gram-positive bacteria known for their pioneering efforts to synthesize a diverse array of valuable secondary metabolites. Actinobacteria are abundantly present in marine environments, where they serve an essential ecological role in recycling refractory biomaterials and producing novel bioactive compounds with pharmaceutical applications [76]. The abundance and distribution of Actinobacteria may vary among the diverse marine organisms. Most actinobacterial species are frequently isolated from bottom sediments, seashores, coastal waters, mollusks, fish, seaweeds, sponges, and mangroves [77,78,79]. According to studies employing culture-dependent and independent molecular methods, abundant novel actinobacterial communities are associated with sponges [80,81,82]. Further, the phylum Actinobacteria are represented by one-sixth of the sponge-associated bacterial sequences in public databases, suggesting that Actinobacteria are a significant phylum among sponge-associated bacteria [83]. Although they are commonly found in bottom sediments, they rarely account for more than 10% of the live microflora.

**Figure 2:**
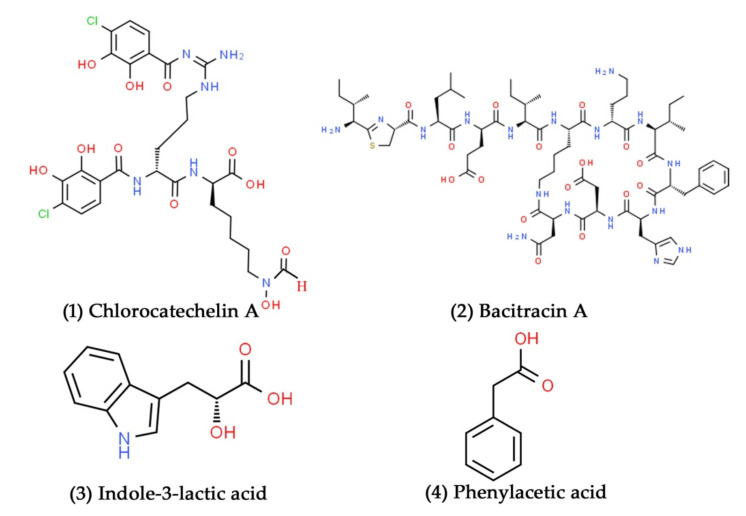
Structural representation of antimicrobial compounds **1**–**4** derived from marine Actinobacteria.

Marine environments are home to many actinobacterial taxa, including *Micromonospora*, *Nocardiopsis*, *Nocardia*, *Plantactinospora*, *Solwaraspora*, *Saccharomonospora*, *Streptomyces*, and *Salinispora* [84]. Among these, *Streptomyces* is a widespread actinobacterial genus found in marine environments. This actinobacterial genus shows a wide range of physiological and metabolic characteristics, including the ability to produce a wide array of extracellular enzymes and secondary metabolites, many of which are exploited by the pharmaceutical industry [85]. Recently, Contreras-Castro et al. isolated 75 Actinobacteria from marine sediment in Punta Arena de La Ventana, Mexico. A total of seventy-one isolates were confirmed belonging to the genus *Salinispora* by 16S rRNA gene identification and multilocus sequence analysis. They subsequently evaluated the antibacterial potential of supernatants from several *Salinispora* strains, revealing that supernatants from ten *Salinispora* strains had excellent growth inhibition activity against ESKAPE pathogens [86]. Similarly, in another study, a total of 148 Actinobacteria were isolated from Diu Island’s intertidal sediments in the Arabian Sea [79]. All the isolates were categorized within actinobacterial taxa: *Glycomycetales*, *Micromonospora*, *Nocardia*, *Nocardiopsis*, *Pseudonocardiales*, and *Streptomyces*. Among the 148 isolates, 62 different actinobacterial isolates were subsequently tested for their antibacterial activity against *Bacillus subtilis*, *Escherichia coli, K. pneumonia*, and *S. aureus*. Out of these, 23 isolates were positive for one or more of the tested bacterial pathogens. Yücel and Yamaç revealed the antibacterial efficacy of *Streptomyces* sp. 1492 against drug-resistant bacterial pathogens, such as *E. faecium*, *S. aureus*, and *A. baumannii* [87]. Chlorocatechelins are novel siderophores with chlorinated catecholate complexes and an acylguanidine structure [88]. Chlorocatechelin A (Figure 2, Compound **1**) extracted from *Streptomyces* sp. inhibited the growth of a wide range of bacterial and fungal pathogens. The new *Streptomyces* sp. MUSC 135T strain was isolated from mangrove forest sediment on Peninsular Malaysia’s east coast. It can produce the broad-spectrum antibiotic molecule bacitracin A (Figure 2, Compound **2**), effective against *S. aureus* ATCC BAA-44 [89,90]. Novel bioactive compounds, such as indole-3-lactic acid (Figure 2, Compound **3**) and phenylacetic acid (Figure 2, Compound **4**), were isolated from the fermentation broth of *Streptomyces* CTF9. In prescreening studies, the bioactive compounds containing *Streptomyces* extract exhibited strong antifungal activity against *C. albicans* and *Mucor miehei* [91].

### 4.2. Antimicrobial Potential of Marine Bacteroidetes

Bacteroidetes is the third most common phylum in the oceans, behind Proteobacteria and Cyanobacteria [92]. Bacteroidia, Cytophaga, Flavobacteria, and Sphingobacteria are the four major classes of the Bacteroidetes phylum. Bacteroidetes is classified into major Gram-negative, rod-shaped, and non-spore-forming bacteria that can live in aerobic and anaerobic environments [93]. Groups of bacteria related to phylum Bacteroidetes may be found in abiotic environments, such as marine sediments, marine seawater, and soil, and biotic habitats, such as animal skin and intestines [94].

A recent comparative genomic analysis revealed abundant Flavobacterium in the marine environment, often associated with fish, marine invertebrates, and algae. According to their genome mining data, the bacteria belong to the genus Flavobacterium have a broad secondary metabolite biosynthesis arsenal, with a high frequency of gene cluster-coding pathways for the production of several pharmacological activities, including antimicrobial ones. Further, they have done antimicrobial phenotypic assays to confirm their genome-derived data [95]. The antimicrobial substances producing marine bacteria belonging to the genus Flavobacterium were isolated from marine invertebrate samples collected from Ancon Bay, Lima, Peru. The bacterial isolates from the genus Flavobacterium exhibited intense antibacterial activity against many human bacterial pathogens [96]. The biotechnological potential of microorganism-derived glucose dehydrogenase enzymes has attracted much interest because of their antimicrobial potential in various industrial applications [97]. The membrane fraction of the marine bacterium *Cytophaga marinoflava* IFO 14170 yielded a new glucose dehydrogenase [98].

### 4.3. Antimicrobial Potential of Marine Cyanobacteria

Cyanobacteria are the only bacterial phylum that derive their energy from the process of photosynthesis. Further, it is the only photosynthetic prokaryote group capable of producing oxygen and absorbing CO_2_ [99]. The marine Cyanobacteria are a powerful nonconventional source of bioactive compounds with a bright and hopeful future in drug development for treating various diseases [100]. Richard E. Moore’s groundbreaking research at the University of Hawaii from the 1970s to the early 2000s shows marine Cyanobacteria to be a potent source of secondary metabolites [101]. Many of these secondary metabolites are distinctively combined with the structural characteristics of peptides and lipids before being further functionalized with atypical oxidations, halogenations, and methylations [102]. Moreover, most of these secondary metabolites are nitrogen-based compounds produced by multimodular nonribosomal polypeptide [100]. The secondary metabolites of marine cyanobacteria have been previously well-reported for their pharmacological activities, such as antibacterial, antiviral, antifungal, anticancer, and antiplasmodium properties [103].

**Figure 3:**
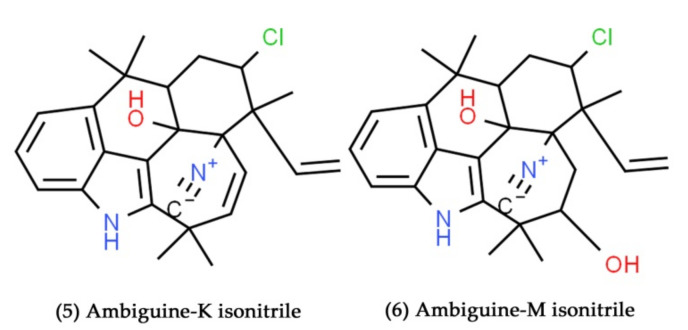
Structural representation of antimicrobial compounds **5** and **6** derived from marine Cyanobacteria.

The secondary metabolites ambiguine-K and M isonitrile (Figure 3, Compounds **5** and **6**), isolated from the marine cyanobacterium *Fischerella ambigua* (UTEX 1903), have exhibited intense antibacterial activity against *Mycobacterium tuberculosis* with MIC values of 6.6 and 7.5 μM, respectively [104]. The novel alkylphenols, anaephenes A-C, were isolated from the cyanobacterium *Hormoscilla* sp. It showed moderate growth inhibition against *S. aureus* [105]. At the concentrations of 22, 6.1, and 22 µg/mL, respectively, anaephenes A, B, and C inhibited the complete visible growth of the *S. aureus*. It seems that there are just a few antiviral drugs that were isolated from marine bacteria when compared to the antibacterial and antifungal drugs. Among them, a maximum of the reported antiviral compounds was derived from marine Cyanobacteria. Glycolipids are found in prokaryotic and eukaryotic photosynthetic organisms, where they are connected with thylakoid membranes. Glycolipids are also found in the heterocystous cell walls of Cyanobacteria [106]. Glycolipids have been found to have distinct antiviral activity in many investigations [107,108]. Gustafson et al. tested extracts of marine Cyanobacteria (*Phormidium tenue* and *Yngbya lagerheimii*) for HIV-1 inhibition using a tetrazolium-based microculture. As a result, sulfonic acid-containing glycolipids were identified as a novel family of HIV-1-inhibiting agents [109]. The novel acylated diglycolipids, and diacylated sulfoglycolipids, were derived from the Cyanobacterium *Oscillatoria raoi* and *Scytonema* sp., respectively. Both of these glycolipids inhibited HIV-1 reverse transcriptase enzymatic activity at a final concentration of 10 µM [106]. The sulfoquinovosylpranosyl lipids from lipophilic extracts of Cyanobacteria were responsible for the strong in vitro suppression of HIV-1 enzymatic activity. With a half-maximal inhibitory concentration (IC50) as low as 24 nM, these sulfolipids effectively and selectively blocked the DNA polymerase activity of HIV-1 reverse transcriptase [108]. Ayehunie et al. demonstrated that an aqueous extract of Cyanobacterium (*Arthrospira platensis*) containing polysaccharides suppressed syncytium formation and HIV-1 replication in human PBMC, T-cell lines, and Langerhans cells at nontoxic concentrations [110]. Calcium spirulan, a sulfated polysaccharide, was isolated from the aqueous extract of *Spirulina platensis* marine blue-green alga. Further, the inhibitory effect of calcium spirulan on a viral replication of measles, HIV-1, herpes simplex virus-1 (HSV-1), mumps, polio, and influenza A was assessed through different cell lines. Calcium spirulan has been shown to impede the reproduction of several enveloped viruses, including HIV-1, HSV-1, influenza A, mumps, and measles. It clearly shows that the calcium spirulan limited the viral penetration into the host cells in a selective manner [111]. Boyd et al. have isolated virucidal protein (cyanovirin-N) from the aqueous cellular extract of Cyanobacterium (*Nostoc ellipsosporum*). The cyanovirin-N inhibited primate retrovirus multiplication (HIV-1 and HIV-2) in vitro at nanomolar concentrations. Cyanovirin-N exerts these antiviral effects through interactions with the viral envelope glycoprotein that seem to be preserved [112].

### 4.4. Antimicrobial Potential of Marine Firmicutes

Firmicutes is a phylum of Gram-positive bacteria. Few have a permeable pseudo-outer membrane that leads them to stain Gram-negative. Due to their ecological variety and unique physiological and molecular features, these bacteria have received much attention in recent decades. They offer a lot of promise for biotechnological applications. *Bacillus* is one of the most important groups of Firmicutes. *Bacillus* sp. has a high-temperature tolerance and can grow quickly in liquid culture. *Bacillus* species are frequently isolated from marine sediment samples and other marine habitats [113,114].

**Figure 4:**
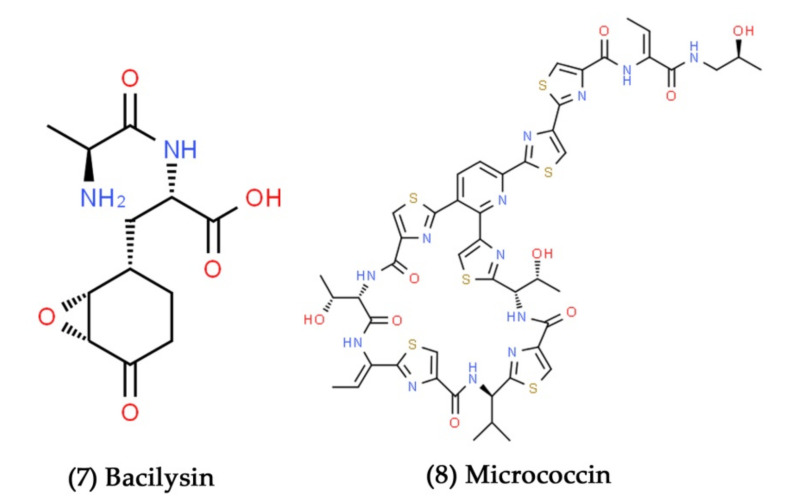
Structural representation of antimicrobial compounds **7** and **8** derived from marine Firmicutes.

Recently, a novel oxatetracyclo ketone antimicrobial compound containing *B. stercoris* MBTDCMFRI Ba37 strain was isolated from estuarine habitats. The isolated compound inhibited the growth of aquatic bacterial pathogens belonging to the genera *Aeromonas* and *Vibrio* [115]. Further, a novel antagonistic secondary metabolite, bacilysin (Figure 4, Compound **7**), was extracted from seaweed-associated *B. amyloliquefaciens* MTCC 10456. It showed selective antifungal activity against *Malassezia globosa* and *M. furfur* [116]. Wang et al. have reported the antibacterial activity of a new thiopeptide-class antibiotic, micrococcin (Figure 4, Compound **8**), isolated from marine-derived *B. stratosphericus*. It showed potent antibacterial activity against Gram-positive bacterial pathogens without considerable cytotoxicity, up to a concentration of 10 µM [117].

### 4.5. Antimicrobial Potential of Marine Planctomycetes

Planctomycetes is a phylum of bacteria with a low frequency of occurrence. This bacterial group is often found in freshwater, soil, and marine environments. Planctomycetes may be free-living or adhere to biotic and abiotic surfaces [118]. Bacteria belonging to the phylum Planctomycetes are critical components of the marine nitrogen and carbon cycles, acting as scavengers after phototrophic blooms [119]. An exploration of Planctomycetes’ genome revealed the possibility of their producing antimicrobial polyketides, nonribosomal peptides, terpenoids, and bacteriocins. According to mass spectrometry studies, the unique bacteria of this group have also produced a new class of antimicrobial compounds [120,121].

In a recent study, stieleriacine, a novel class of N-acylated tyrosines, was isolated from the marine Planctomycete strain *Stieleria maiorica* Mal15^T^. It reduced the growth and biofilm of the producing strain, *S. maiorica* Mal15^T^, and the co-occurring marine bacterial species, respectively [122]. Similarly, in another study, N-acylated dehydrotyrosine derivative compound, namely, stieleriacine D, was isolated from marine Planctomycete strain *S. neptunia* sp. nov. It inhibited the growth of *S. aureus* and *Micrococcus luteus* at the concentrations of 16.7 and 66.7 μg/mL, respectively [119].

### 4.6. Antimicrobial Potential of Marine Proteobacteria

Proteobacteria is the most diverse phylum of Gram-negative bacteria. The group is split into six classes, some identified by Greek letters spanning from Alpha to Epsilon, while others are known as Oligoflexia and Acidithiobacillia [70]. Previous research has shown that Proteobacteria may dominate microbial communities, even in deep-sea environments [123,124]. The marine bacterial phyla Proteobacteria are frequently reported as major bioactive chemical producers in marine macro-organisms. They can produce biologically active molecules with antibacterial, antiviral, antibiofilm, antifouling, and anticancer activities [125].

**Figure 5:**
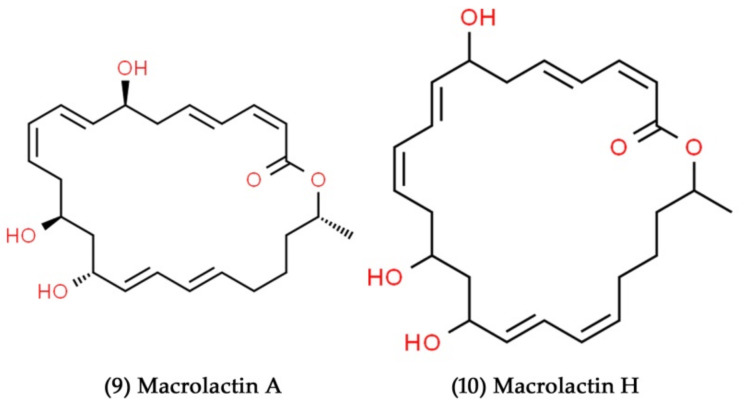
Structural representation of antimicrobial compounds **9** and **10** derived from marine Proteobacteria.

The *Pseudoalteromonas* species, associated with marine invertebrates and seaweed, have been identified as producers of the enormously potent antibiotic molecule, thiomarinol [126]. Recently, Dat et al. have examined the diversity and antimicrobial activity of cultivable bacteria in Vietnamese sponges. A total of 460 cultivable bacterial isolates were isolated from 18 marine sponges, in which nearly 58% of the bacteria belonged to the phylum Proteobacteria. Moreover, they have identified secondary metabolites, such as macrolactin A (Figure 5, Compound **9**), and macrolactin H (Figure 5, Compound **10**), as the major bioactive compounds. The isolated compounds exhibited strong antibacterial actions against a wide range of pathogenic organisms [127].

## 5. Antimicrobial Potential of Marine Sediment-Derived Bacteria

Since the 1940s, the soil has been viewed as the primary source for isolating antibiotic compounds. A shift from soil to other sources, such as marine sediment, has been seen in recent decades [128]. Sediment refers to any solid particle of inorganic or organic materials. Marine sediments include those found near the shore, such as rocks and cobbles, seashell fragments, and sand, and in the mud at the bottom of ocean waters. Furthermore, the continental and oceanic crust, volcanoes, chemical processes, outer space, microorganisms, and plants and animals, all influence the formation of marine sediments [129]. Marine sediments may be classified into two types based on their source area: near-shore sediments and deep-sea sediments. Numerous bacterial strains isolated from near-shore sediments are closely related to strains previously identified in soil [130]. This is because, unsurprisingly, many studies on the isolation of marine bacterial isolates from near-shore sediments have yielded the known secondary metabolites. As a result, access to marine deep-sea sediments has become a priority, increasing the probability of discovering novel marine bacterial isolates [70,128].

**Figure 6:**
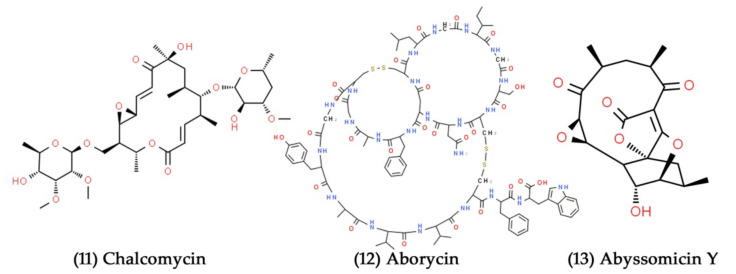
Structural representation of antimicrobial compounds **11**–**13** from sediment-derived marine bacteria.

Macrolactins are 24-membered lactone natural products with several pharmacological activities, making them attractive therapeutic candidates. They are mainly generated by marine-derived microorganisms [128]. A Macrolactin class of antibacterial and antiviral drugs were isolated from unclassified deep-sea sediment marine bacteria. Among the different macrolactins isolated, macrolactin A showed potent antibacterial activity against *S. aureus* and *B. subtilis*. Additionally, macrolactin A was an effective inhibitor of the HSV type-1 (strain LL) and type-2 viruses (strain G), with IC50 values of 5.0 and 8.3 µg/mL, respectively [129]. Marine *Streptomyces* sp. B7064 was isolated in mangrove sediment in Pohoiki, Hawaii, and was found to produce chalcomycin (Figure 6, Compound **11**), a novel macrolide antibiotic. The compound exhibited potent antibacterial activity against *S. aureus*, *E coli*, and *B. subtilis*, with minimum inhibitory concentration (MIC) values of 0.39, 750, and 6.25 µg/mL, respectively [131]. Aborycin (Figure 6, Compound **12**) was extracted and identified from the *Streptomyces* sp., which was isolated from the deep-sea sediments of the South China Sea [132]. With MIC values ranging from 8.0–128 µg/mL, the compound showed moderate bacteriostatic activity against different strains of *S. aureus*. Further, this secondary metabolite inhibited *E. faecalis* and *B. thuringiensis* at the MIC values of 8.0 and 2.0 µg/mL, respectively. A novel polyketide compound abyssomicin Y (Figure 6, Compound **13**) was isolated from the marine-sediment-derived Verrucosispora strain MS100137, which exhibited potent antiviral activity against the influenza A virus at a concentration of 10 μM [133].

**Figure 7:**
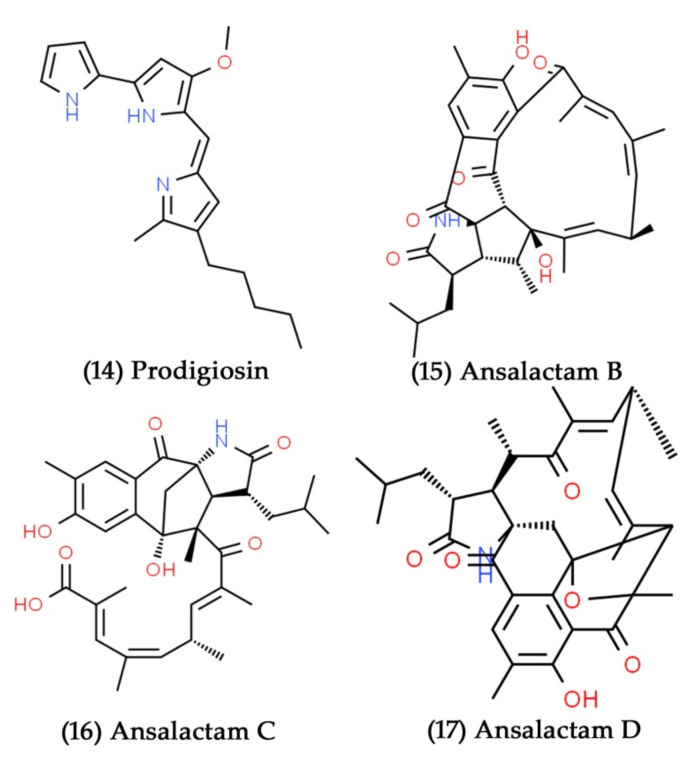
Structural representation of antimicrobial compounds **14**–**17** from sediment-derived marine bacteria.

Certain marine bacteria have great potential for producing varied and remarkable characteristics, such as pigment production [134]. These marine-pigmented bacteria have been shown to possess various biological functions, including antibacterial, anticancer, antifungal, and immunosuppressive functions [135]. The red-pigmented prodigiosin compound was first isolated and identified as a secondary metabolite from the ubiquitous bacteria *Serratia marcescens* [136]. Apart from *S. marcescens*, many marine bacteria belonging to the genera *Actinomadura*, *Pseudoalteromonas*, *Pseudomonas*, and *Streptomyces* have been reported to produce red-pigmented prodigiosin and related compounds [137,138,139]. The *Hahella* sp. isolated from the sediment sample from Nagasaki Prefecture, Japan, secreted enormous amounts of red-pigmented prodigiosin (Figure 7, Compound **14**), which showed excellent antibacterial activity against different strains of *S. aureus* [140]. The polyketide ansalactams A–D are radically modified ansamycins that were obtained from the marine sediment culture of *Streptomyces* sp. CNH189. The antibacterial activity of ansalactams B, C, and D (Figure 7, Compounds **15**–**17**) against *S. aureus* was moderate, with MICs of 31.2, 31.2, and 62.5 µg/mL, respectively. In contrast, ansalactam A was inactive up to 100 µg/mL [141].

**Figure 8:**
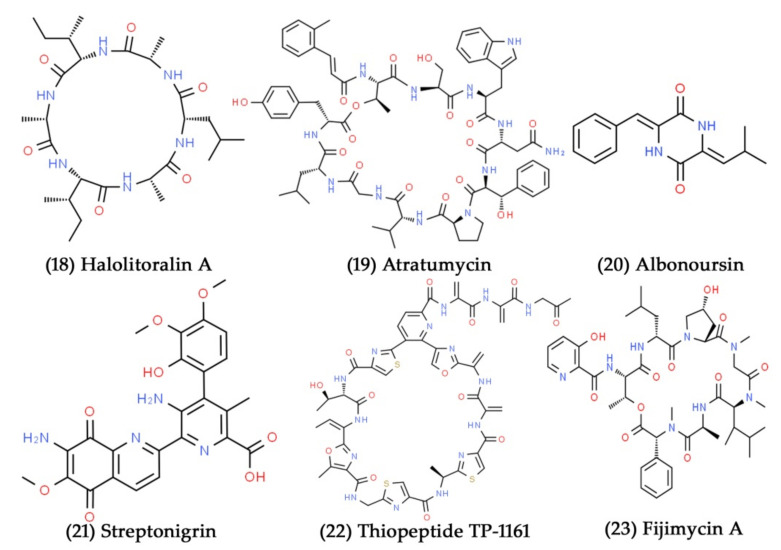
Structural representation of antimicrobial compounds **18**–**23** from sediment-derived marine bacteria.

Marine bacteria are the major source of structurally distinct and biologically significant nonribosomal peptides, particularly cyclopeptide derivatives. Yang et al. isolated three cyclopeptides derivatives, the halolitoralins A, B, and C, from the marine-sediment-derived *Halobacillus litoralis* YS3106 strain. In comparison to the other two cyclopeptides, halolitoralin A (Figure 8, Compound **18**) has efficient antifungal activity against *Tricophyton rubrum* and *C. albicans* at the concentrations of 25 and 20 µg/mL, respectively [142]. Atratumycin (Figure 8, Compound **19**) is a cyclic dipeptide that exhibits antibacterial action against *M. tuberculosis*, with MIC values of 14.6 and 3.8 µM against the *M. tuberculosis* H37Rv and H37Ra strains, respectively. It was isolated from an extract of *S. atratus* from the deep-sea sediments of the South China Sea [132]. *Streptomyces* sp. SMS636 extract yielded albonoursin (Figure 8, Compound **20**), and streptonigrin (Figure 8, Compound **21**), recovered from deep-sea sediments in the South China Sea. With MIC values of 12.5 and 0.78 µg/mL, respectively, albonoursin and streptonigrin exhibited substantial antibacterial activity against *S. aureus* [143]. Engelhardt et al. isolated eighteen actinomycete strains from the marine sediment sample collected in the Trondheim Fjord, Norway. Among the eighteen actinomycete extracts, a total of 4 and 10 actinomycete fermentation extracts substantially inhibited the growth of *C. albicans* ATCC 10231 and *M. luteus* ATCC 9341, respectively. They then purified and identified novel thiopeptide antibiotic TP-1161 (Figure 8, Compound **22**) from the active extracts [144]. In another study, the novel depsipeptide antibacterial compound, fijimycin A (Figure 8, Compound **23**), was isolated from marine sediment-derived *Streptomyces* sp. and showed profound antibacterial activity against different strains of *S. aureus* with MIC values between 4 and 16 μg/mL [145].

**Figure 9:**
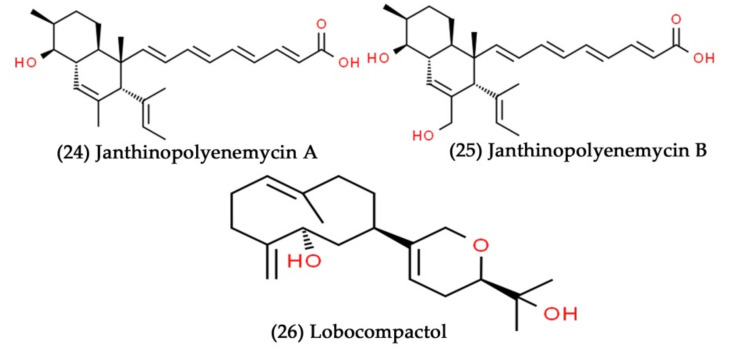
Structural representation of antimicrobial compounds **24**–**26** from sediment-derived marine bacteria.

The cocultivation of two or more microorganisms has gained increased attention due to the possibility of finding novel bioactive natural compounds [146]. The cocultivation of marine sediment-derived bacterial strains, such as *Janthinobacterium* sp. ZZ145 and ZZ148, induced the production of two novel polyketides, including janthinopolyenemycins A and B (Figure 9, Compounds **24** and **25**). Furthermore, both janthinopolyenemycins A and B exhibited excellent antifungal activity against *C. albicans* with a MIC value of 15.6 µg/mL [147]. In another study, Cho et al. isolated the actinomycete strain (*S. cinnabarinus* PK209) from marine sediment collected from the coast of Korea. They cocultivated the *S. cinnabarinus* PK209 with *Alteromonas* sp. to induce the production of the diterpene compound lobocompactol (Figure 9, Compound **26**). Then, the overproduced lobocompactol was tested for their growth inhibitory potential. The obtained data revealed that lobocompactol acted as an antifouling agent on macroalga *Ulva pertusa* by preventing the attachment and growth of different fouling bacteria [148].

## 6. Antimicrobial Potential of Marine Water-Derived Bacteria

**Figure 10:**
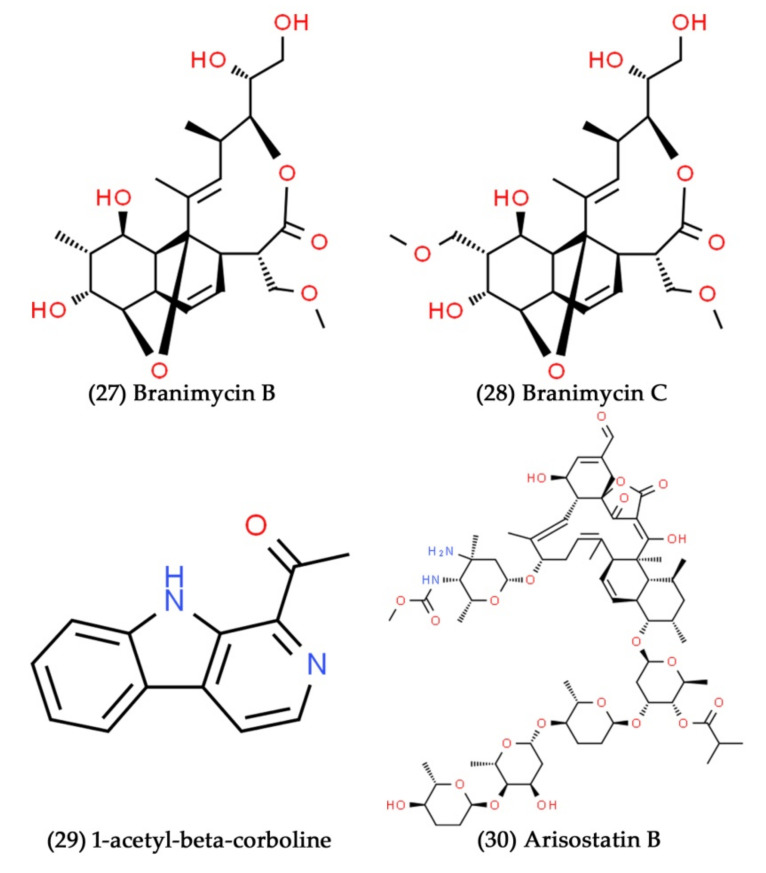
Structural representation of antimicrobial compounds **27**–**30** from water-derived marine bacteria.

Microbial life in marine water is diverse, with bacteria, viruses, fungi, and spores being the most prevalent [40]. Novel antibacterial dipeptide compounds, such as unnarmicins A and C, were extracted from marine bacterium *Photobacterium* sp. MBIC06485. This marine bacterial strain was isolated from the coastal seawater collected from Onna Beach, Okinawa, Japan. The isolated dipeptide compounds selectively inhibited the two marine environmental pathogenic strains, *Alphaproteobacteria* and *Pseudovibrio* [149]. A bioassay-guided fractionation of a fermentation broth of *Pseudonocardia carboxydivorans* M-227, isolated from deep-seawater in the Avilés submarine canyon, disclosed the presence of two antibiotic molecules, branimycins B and C (Figure 10, Compounds **27** and **28**). These two antibiotic molecules exhibited moderate to substantial antibacterial activities against different Gram-positive and Gram-negative bacterial pathogens [150]. The marine bacteria *Pseudomonas* UJ-6, isolated from the seawater sample, has been shown to possess the secondary metabolite 1-acetyl-beta-corboline (Figure 10, Compound **29**). Further, the bioactive compound extracted from *Pseudomonas* UJ-6 had antibacterial action against *S. aureus* with the MIC range of 32–128 µg/mL [151]. Arisostatin B (Figure 10, Compound **30**), a novel tetroarcin antibiotic, was extracted from the cultivated broth of the *Micromonospora* sp. obtained from sea water in Toyama Bay, Japan. At concentrations of 3.1, 25, and 50 µg/mL, respectively, arisostatin B inhibited the growth of *M. luteus*, *B. subtilis*, and *S. aureus* [152]. Arena et al. extracted the exopolysaccharide (EPS) from *B. licheniformis*, isolated from shallow marine water from the hot spring on Vulcano Island, Italy. The extracted EPS potentially enhanced the innate immunity of human peripheral blood mononuclear cells (PBMC) against the herpesvirus [HSV-1] infection by promoting Th1-type cytokine polarization [153]. Furthermore, the same research group extracted EPS from the marine *Geobacillus thermodenitrificans*, isolated from the deep-sea vent of Vulcano Island, Italy. They found that the EPS treatment inhibited HSV-1 multiplication in PBMC by elevating the amounts of IL-12, IL-18, TNF-, and IFN- [154].

In addition to these, several marine bacterial isolates derived from marine sediments and water have been well-reported for their antimicrobial activities, which are tabulated in Table 1.

## 7. Marine Fauna

Marine fauna is extremely diverse and encompasses living organisms from microscopic zooplankton to blue whales. The marine fauna classification includes all animals and living organisms present in the ocean, regardless of their size. They play critical roles in maintaining key ecological processes and functions within the marine ecosystem. Apart from these, certain marine fauna act as a treasure house for synthesizing several novel bioactive compounds. The following sections intensely discuss the antimicrobial potentials of marine bacteria associated with certain important marine fauna, such as sponges, corals, and mollusks.

### 7.1. Antimicrobial Potential of Bacteria Associated with Marine Sponges

Marine sponges contribute significantly to benthic communities worldwide, both in biomass and their ability to affect benthic or pelagic processes [197]. Sponges are abundant in tropical reefs, but they also inhabit polar regions, the deep sea, and freshwater lakes and rivers. They are one of the oldest multicellular invertebrates and exist in a broad range of colors and textures. To date, over 8000 sponge species have been identified in various marine and freshwater environments [198]. Marine sponges are habitat to a diverse array of microbial communities, including bacteria, fungi, archaea, and viruses, which provide an essential source of natural products. Microbial biomass may take up to 35% of the sponge’s volume. These sponges are classified as “high-microbial abundance”. The true origin of several sponge-derived secondary metabolites remains unclear to date. Secondary metabolites may be generated by sponges, their microbial symbionts, or the interactions between sponges and symbionts [199]. Interestingly, many studies have shown that several bioactive compounds discovered in sponges may be of bacterial origin, according to the chemical structural resemblance to those found in terrestrial and marine microorganisms [197,200]. It suggests that sponge-associated bacteria, rather than the host, are the real producers. Furthermore, these symbiotic bacteria also play a significant role in the health and survival of marine sponges [201]. As a result, many researchers concentrate their efforts on sponge-associated bacteria, which may be used to screen and isolate bioactive compounds.

**Figure 11:**
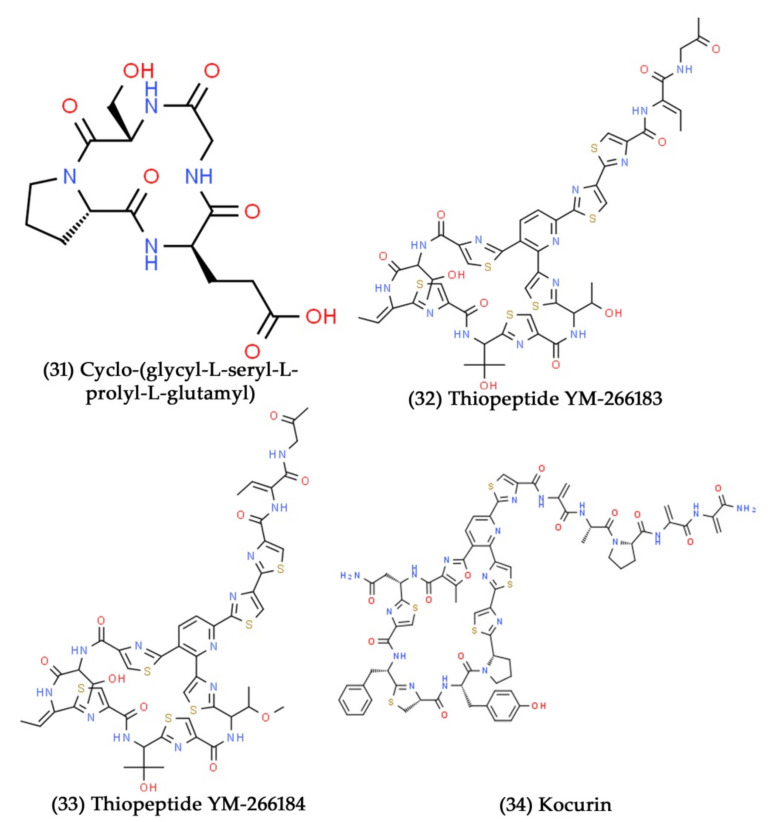
Structural representation of antimicrobial compounds **31**–**34** derived from bacteria associated with marine sponges.

Recently, Altuğ et al. isolated the sponge-associated bacteria collected from the Sea of Marmara, Turkey. They then assessed the antibacterial potential of the methanolic extract of marine sponge-associated bacteria against different bacterial pathogens, such as *S. aureus*, *Vibrio vulnificus*, and *E. coli*. At the tested concentrations (7.8 to 1000 µg/mL), the extracts significantly inhibited the growth of all the tested bacterial pathogens. Finally, they identified this efficient sponge-associated bacterium related to *B. cereus* through molecular identification [202]. The majority of actinomycetes obtained from marine sources are believed to have originated from marine sponges, with *Streptomyces* being the most common species [203]. Numerous studies have shown that the isolation of several novel bioactive compounds from *Streptomyces* is associated with marine sponges [204,205,206]. Around 22% of the bioactive compounds are isolated from marine sponges associated with actinomycetes. In a recent study, Fahmy et al. have isolated the antimicrobial compounds from *Streptomyces* sp. associated with the marine sponge collected from the Egyptian Red Sea coast [62]. In another recent work, a novel antiviral compound, namely homoseongomycin, was isolated from marine sponge-associated actinomycete bacteria K3-1. It showed potent antiviral activity against alphaviruses, such as the eastern and Venezuelan equine encephalitis viruses, with 50% effective concentrations of 1.2 and 8.6 μM, respectively [207]. Mitova et al. isolated a novel cyclopeptide compound, namely, cyclo-(glycyl-l-seryl-l-prolyl-l-glutamyl) (Figure 11, Compound **31**) from the extract of marine Agrobacterium (*Ruegeria* strain) associated with the sponge *Suberites domuncula*. The isolated cyclopeptide has exhibited potent antibacterial activity against *B. subtilis* at the concentration of 25 µg/mL [208]. In another study, novel thiopeptide antibiotic substances, namely, YM-266183 and YM-266184 (Figure 11, Compounds **32** and **33**), were isolated from marine bacteria *B. cereus* QN03323 associated with the sponge *Halichondria japonica*. Further, both of these thiopeptide antibiotic substances have shown excellent growth inhibitory potential against Gram-positive bacterial pathogens [209]. A thiazolyl peptide derivative compound, kocurin (Figure 11, Compound **34**), was extracted from the culture broth of *Kocuria palustris*. This bacterial strain was isolated from a marine sponge sample collected in the Florida Keys, United States of America (USA), and the compound kocurin showed excellent antibacterial and antifungal activities against certain bacterial and fungal pathogens [210].

**Figure 12:**
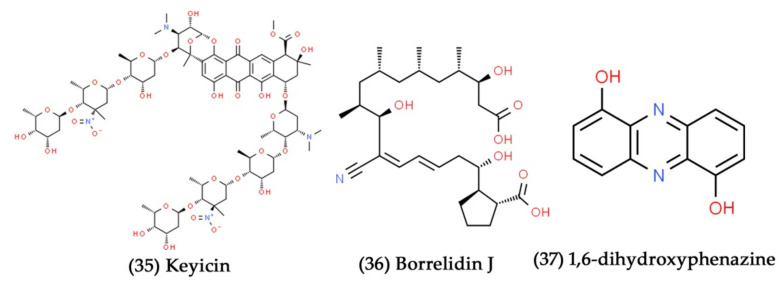
Structural representation of antimicrobial compounds **35**–**37** derived from bacteria associated with marine sponges.

The cocultivation of bacterial isolates obtained from marine sponges, such as *Rhodococcus* sp. and *Micromonospora* sp., stimulated the synthesis of a novel antibiotic molecule, keyicin (Figure 12, Compound **35**). Further, the extracted bioactive compound keyicin selectively inhibited the growth of Gram-positive bacterial pathogens, especially *Mycobacterium* sp. and *Rhodococcus* sp. [211]. Similarly, the coculture of actinomycete (*S. rochei* MB037) derived from a marine sponge (*Dysidea arenaria*) with fungus (*Rhinocladiella similis* 35) stimulated the production of borrelidin J (Figure 12, Compound **36**). The extracted fatty acid compound borrelidin J exhibited profound antibacterial action against *S. aureus* with a MIC value of 0.195 μg/mL [212]. In another study, the cocultivation of two marine sponge-derived actinomycetes, *Nocardiopsis* sp. RV163 and *Actinokineospora* sp. EG49, induced the production of ten different secondary metabolites related to β-carboline, diketopiperazine, and angucycline derivatives. Among them, the prenylated derivative compound 1,6-dihydroxyphenazine (Figure 12, Compound **37**) only showed antibacterial activity against *Bacillus* sp. P25 [213].

### 7.2. Antimicrobial Potential of Bacteria Associated with Marine Corals

Corals, among marine organisms, are potential producers of marine bioactive compounds and have received a lot of attention. Deep-sea corals produce a variety of bioactive natural products with structurally diverse properties [214]. Microorganisms, primarily bacteria, have been shown to colonize different sections of coral tissues (gastrovascular cavity, mucus layer, and skeleton) and to have a role in coral development, health, and stress tolerance [215]. Further, earlier studies show that the coral microbiome protects coral hosts by producing antimicrobial agents, suppressing pathogenic metabolic enzymes, interrupting cell-to-cell communication systems, and competitively eliminating pathogens from the host cell surfaces [216,217,218,219]. Many structurally distinct bioactive compounds with a wide variety of pharmacological activities, including antimicrobial compounds, may be produced by coral-associated bacteria against a wide range of pathogenic organisms [218].

**Figure 13:**
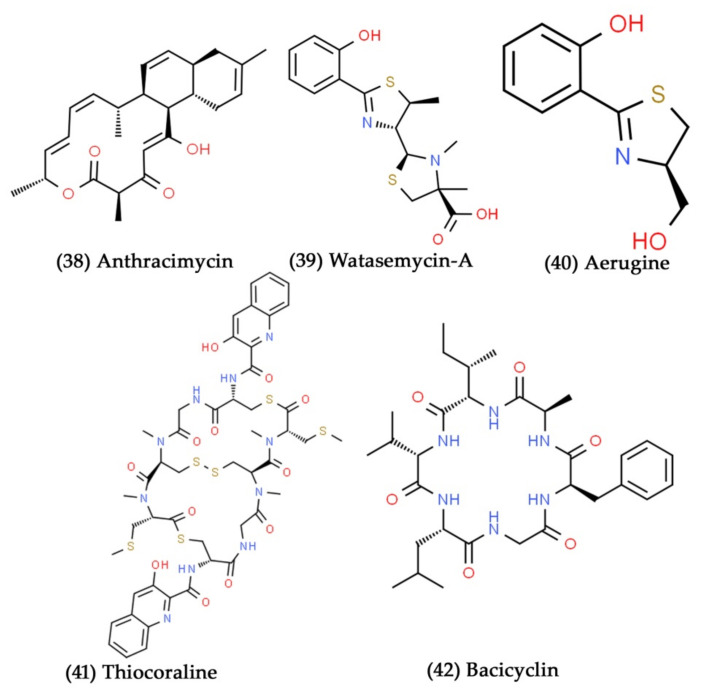
Structural representation of antimicrobial compounds **38**–**42** derived from bacteria associated with marine corals and mollusks.

Rodríguez et al. have isolated the strain *S. cyaneofuscatus* M-169 from a gorgonian coral obtained in the deep-sea water of Avilés Canyon, Cantabrian Sea. They then extracted the polyketide compound, anthracimycin (Figure 13, Compound **38**), from the acidified ethyl acetate extract of *S. cyaneofuscatus*. It showed potent antibacterial activity against Gram-positive bacterial pathogens, such as *S. aureus*, *E. faecalis*, and *E. faecium*, at the concentration of 0.03 μg/mL [220]. Watasemycin-A, aerugine, and pulicatin-G are three thiazole derivatives extracted from *Streptomyces* sp. OUCMDZ-1703, a coral-associated actinomycetes strain. Watasemycin-A (Figure 13, Compound **39**) and aerugine (Figure 13, Compound **40**) were the first compounds that exhibited strong antibacterial action against different clinical strains of *S. aureus* with the MIC value of 7.81 μg/mL [221]. Sarmiento-Vizcaíno et al. collected 87 deep-sea coral reef samples from the submarine Avilés Canyon, Spain. They then isolated 18 different cultivable Actinobacteria from the coral samples and tested their antimicrobial potential. The obtained results revealed that the isolated deep-sea Actinobacteria exhibited both antibacterial and antifungal activities [222]. The strain *Micromonospora marina* was isolated from a soft coral and produced a novel depsipeptide compound, namely, thiocoraline (Figure 13, Compound **41**). With a MIC value of 0.05 µg/mL, thiocoraline exhibited strong antibiotic action against Gram-positive bacterial pathogens, such as *B. subtilis*, and *S. aureus* [223].

### 7.3. Antimicrobial Potential of Bacteria Associated with Marine Mollusks

Mollusks are the largest marine phylum, accounting for 23% of marine organisms. Mollusks with shells are assumed to be low in secondary metabolites, although they may produce peptide toxins for both defense and predation [70]. Secondary metabolites help to protect mollusks even without shells. The systematic structural analysis of certain secondary metabolites has indicated that these small chemical compounds are produced by symbiotic bacteria, not by mollusks [224].

Bacicyclin (Figure 13, Compound **42**), a novel cyclic hexapeptide, was identified from *Bacillus* sp. BC028. This marine bacterial strain was isolated from the common mussel (*Mytilus edulis*). It has antibacterial properties against *S. aureus* and *E. faecalis*, with MIC values of 12 and 8 µM, respectively [225]. *S. sampsonii* SCSIO 054 is a marine-gastropod-mollusk-associated bacteria with a biosynthetic gene cluster that governs the synthesis of the antibacterial compound julichromes [226]. The Hawaiian bobtail squid contains symbiotic bacterial consortia in the auxiliary nidamental gland (a female reproductive system that shields eggs from fouling microorganisms). Nineteen bacterial isolates were isolated from these bacterial consortia. Further, these isolates were tested for their ability to inhibit the growth of Gram-positive and Gram-negative bacterial pathogens. Among these, the two bacterial isolates, *Pseudoalteromonas* sp. JC28 and *Leisingera* sp. ANG59, showed potential antibacterial activity against tested bacteria [227].

In addition to these, the antimicrobial activity of numerous bacteria associated with different marine fauna has been extensively documented, as shown in Table 2.

## 8. Marine Flora

Like marine fauna, marine flora is also abundant in nature. Some are so tiny and can only be seen with the aid of a microscope. They have various living forms; some float, while others are attached to different surfaces with a holdfast. Organisms in marine flora are rich in minerals and provide nutraceuticals that can be added as the necessary components of the diet. In general, the host-microbe interaction plays a crucial role in host adaptation to different environments [46]. This kind of microbe interaction, especially with plants, results in the production of novel compounds with potential applications in several areas, such as medicine, environmental protection, bioremediation, and others [47]. Hence, the following sections describes the potential antimicrobial compounds from bacteria associated with certain marine flora, such as seaweeds, seagrasses, and mangroves.

### 8.1. Antimicrobial Potential of Bacteria Associated with Marine Seaweeds

Seaweeds (macroalgae) are photosynthetic organisms with an essential role in sustaining the marine ecosystem. Seaweeds are divided into three types based on their pigmentation. They are classified as green algae, brown algae, and red algae. Brown seaweeds contain fucoxanthin and chlorophyll a and c pigments, red seaweeds contain allophycocyanin, phycoerythrin, xanthophylls, and chlorophyll-a pigments, and green seaweeds contain xanthophylls and chlorophyll a and b pigments [244,245]. The seaweed surfaces offer an ideal substrate for microorganisms to colonize, and for various secret organic compounds that serve as nutrients for microorganisms to proliferate. Therefore, they provide an excellent habitat for several microorganisms [246]. Numerous studies have shown that seaweed-associated bacteria are essential for determining the host’s basic morphology, growth, and development [40,76]. Seaweeds may also be viewed as a source of microbial nutrients, which results in intense competition between diverse microbial populations [66]. Moreover, seaweeds provide nutrients for associated microorganisms which, in turn, defends them from biological threats, such as pathogenic and fouling organisms, via the synthesis and release of bioactive substances [247]. Microbial communities that live on the surfaces of seaweeds are very complex and dynamic, consisting of a diverse array of microorganisms, such as bacteria, fungi, protozoa, and diatoms [248,249]. Numerous investigations have shown that bacteria associated with seaweeds are unique from bacteria present in seawater. These bacteria mostly come under the phyla of Actinobacteria, Bacteroidetes, Firmicutes, Planctomycetes, and Proteobacteria [250,251].

**Figure 14:**
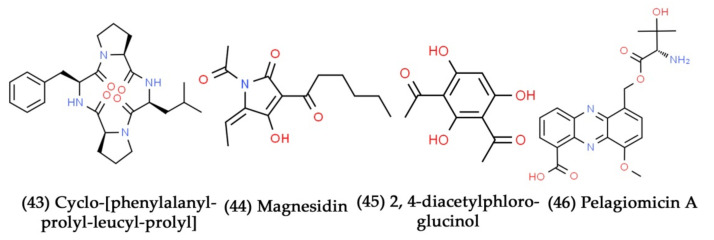
Structural representation of antimicrobial compounds **43**–**46** derived from bacteria associated with marine seaweeds.

A new cyclic tetrapeptide compound, namely, cyclo-[phenylalanyl-prolyl-leucyl-prolyl] (Figure 14, Compound **43**), was identified from marine bacteria *Pseudomonas* sp. associated with the seaweed (*Diginea* sp.) that had excellent growth inhibitory potential against *V. anguillarum* and *B. subtilis* [252]. Franks et al. isolated the marine bacterium *P. tunicata* from the surface of *U. australis*. The antifungal compounds produced by *P. tunicata* provide a competitive edge over marine fungi during surface colonization. Further, tambjamine is a yellow-pigmented chemical molecule from *P. tunicate* that seems to have antifungal activity [253,254]. The novel magnesium-containing antibiotic molecule, magnesidin A (Figure 14, Compound **44**), was isolated from the marine bacterium *P. magnesiorubra* associated with the seaweed *Caulerpa peltata*. It selectively inhibited the growth of *Staphylococcus* sp. and *Bacillus* sp. [255]. Another bioactive compound, namely, 2, 4-diacetylphloroglucinol (Figure 14, Compound **45**), was isolated from a novel *Pseudomonas* sp. AMSA. This surface-associated bacterial strain was isolated from the *Ceratodyction spongiosum* and significantly hindered *S. aureus* growth at the concentration of 4 mg/L [256]. Ravisankar et al. identified an alkaloid compound from *Pseudomonas* sp. associated with the seaweed *Padina tetrastromatica*. At a dosage of 300 µg, this bioactive compound inhibited the growth of Gram-negative bacterial pathogens, such as *P. aeruginosa* and *K. pneumoniae*, with an inhibitory zone of 10 and 15 mm, respectively [257]. *Pelagiobacter variabilis*, a novel marine bacterium, was isolated from the macroalga *Pocockiella variegata*. This bacterium produces a pelagiomicin A to C series of chemical molecules. Pelagiomicin A (Figure 14, Compound **46**) showed potent antibacterial activity against a wide range of Gram-positive and Gram-negative bacterial pathogens [258].

### 8.2. Antimicrobial Potential of Bacteria Associated with Marine Seagrasses

Seagrasses provide major habitats for marine microorganisms and have a primary ecological function in coastal regions worldwide. Seagrasses are a diverse species of angiosperms found in the ocean along with macroalgae [259]. Generally, the marine habitat is often used as a nursery by various fish species [260]. The direct contact between animal and plant cells has been shown to promote metabolic pathways in microbes associated with seagrasses. Further, microbiological investigations confirm this theory by demonstrating that the microbial activity of the seagrass-associated holobiont is greater than that of the microbial community grown in a nonvegetated region [251]. Seagrasses have a large proportion of secondary metabolites, such as fatty acids and polyphenols, which are essential for adaptation to biotic and abiotic habitats, as well as the defense mechanism [261]. Moreover, microorganisms associated with seagrasses may preserve their environment by producing bioactive compounds that defend against biological threats, such as pathogenic and fouling organisms. For the same reason, they were recognized as a source of novel bioactive compounds in abundance [262].

A total of 162 rhizome and endophytic bacteria isolates were enumerated from marine seagrass *Halodule uninervis* collected from the coastal zone of Jeddah, Saudi Arabia. In an in vitro assay, antifungal screening of isolated bacteria showed 19 strains capable of inhibiting the growth of four pathogenic fungi: *Rhizoctonia solani*, *Phytophthora capsici*, *Pythium ultimum*, and *Pyricularia oryzae* [263]. A recent study reported the isolation and genotypic identification of marine epiphytic bacteria with potent antibacterial activity from the Kuicheai seagrass (*H. uninervis*). *Bacillus*, *Oceanimonas*, *Paenibacillus*, and *Planomicrobium* were among the four genera of epiphytic bacteria identified. Using the perpendicular streak technique, *Oceanimonas* sp. PSS 241 was shown to have effective antibacterial activity against pathogenic *S. aureus* [264].

### 8.3. Antimicrobial Potential of Marine Bacteria Associated with Mangroves

Mangroves occupy 60–70% of the world’s tropical and subtropical coastlines. This unique environment is a habitat for a diverse array of microorganisms. Microorganisms associated with mangroves may withstand certain changes in physicochemical environments, particularly in high-saline conditions [265]. As a result, they are critical components of this marine ecosystem. Apart from their critical function in ecological balance maintenance and biogeochemical processes, mangrove-associated microbes have a higher biotechnological potential in various fields, including pharmaceutical industries [251]. Marine microorganisms thrive in the lower portion of trunks and the aerating roots of mangrove plants that are permanently or intermittently immersed in saltwater. On the other hand, terrestrial microorganisms are often found in the upper portions of mangrove-aerating roots and trunks [266].

The bacteria associated with mangroves, particularly Actinobacteria, are a unique and neglected source of natural pharmaceutical products. There is substantial data from diverse mangrove forests worldwide indicating Actinobacteria’s widespread distribution and enormous promise in the pharmaceutical sector [251,267]. The presence of actinomycetes has been well-described in a variety of mangrove environments around the world. In an earlier study, 23 actinomycete species were identified in the Pichavaram mangrove, India, most assigned to the genus *Streptomyces* [268]. In another study, a total of 518 *Streptomyces* isolates were identified in mangrove habitats near Porto Novo, India, by Laksmanaperumalsamy et al. [269]. Eccleston et al. revealed the wide spread of actinomycetes belonging to the genus *Micromonospora* in the mangrove plants of Australia’s Sunshine Coast [270]. Similarly, Xie et al. and Huang et al. reported the isolation of a rifamycin-producing *Micromonospora* from mangroves in the South China Sea [271,272]. Retnowati identified an actinomycete, *Streptomyces*sp., in mangrove soil on the eastern coast of Surabaya, Indonesia. It inhibited the growth of both Gram-positive and Gram-negative bacterial pathogens [273]. Santhi and Jebakumar reported that *Streptomyces* sp. isolated from mangrove sediment had profound antibacterial activity against *S. aureus* and *Salmonella typhi* [274].

Likewise, as shown in Table 3, the antimicrobial activity of several bacteria associated with different marine flora has been comprehensively documented.

## 9. Molecular Approaches for the Identification and Development of Novel Antimicrobial Agents from Marine Bacteria

Modern advancements in genome sequencing have revealed that microorganisms could produce much more structurally diverse secondary metabolites, owing to several putative biosynthetic gene clusters (BGCs) that encode for secondary metabolites that are not observed under conventional culture conditions [294]. BGCs are composed of a group of genes that collectively code for the synthesis of one or more specific metabolites [295]. These clusters are needed to make a wide range of structurally varied compounds, such as polyketides and nonribosomal peptides. Several methods have been developed in the past decade to trigger these cryptic biosynthetic pathways and stimulate the synthesis of novel secondary metabolites from microorganisms [296]. Polyketide synthases (PKS) and nonribosomal peptide synthases (NRPS) are two examples of complex enzymatic machinery that have been shown to be responsible for the synthesis of marine bacterial bioactive secondary metabolites. Recently, Konstantinou et al. confirmed the potential of sponge-associated Cyanobacteria extracts from various taxonomic groups to produce antibacterial activity against *S. aureus* [297]. Moreover, they have performed phylogenetic analysis and molecular screening for genes encoding PKS and NRPS on sponge-associated Cyanobacteria. The obtained data reveal that the genes responsible for PKS were more ubiquitous than those responsible for NRPS. Graça et al. performed the molecular and bioactivity assays to evaluate the antimicrobial production of a vast and varied collection of Planctomycetes derived from marine macroalgae. Molecular analysis revealed that 95 percent of derived Planctomycetes contained one or both secondary bioactive genes: the NRPS and PKS genes. Moreover, the bioactivity assays revealed a large number of Planctomycetes (*Planctomyces brasiliensis*, *Rhodopirellula lusitana*, *R. baltica*, and *R. rubra*) with bioactive extracts that exhibited antibacterial and antifungal activities against *B. subtilis* and *C. albicans*, respectively [298].

## 10. Future Research Directions

According to scientific evidence, around 2% of bacteria on Earth can be readily cultivated through a culture-dependent method [299]. There are presently 61 different bacterial phyla identified, 31 of which have no cultivable representatives. Thus, most bacteria are uncultivable and, therefore, the conventional culture techniques always underestimate the richness of diverse bacterial communities [300]. As a result of the development of certain culture-independent methods, there has been a shift toward the characterization of mixed bacterial communities within marine environmental biomass. However, several advanced molecular techniques should be developed for isolating the bacteria from marine environments, which may open new avenues to discovering more structurally unique and effective antimicrobial compounds. The genome study, functional gene screening, and gene manipulation of marine bacteria reveal the potential for producing a wide variety of novel antimicrobial compounds [301,302,303]. However, only a tiny percentage of the bacterial genes were expressed under certain cultivation states [304]. As a result, distinctive methods should be taken to activate the gene transcription and metabolic reprogramming efficiently. The extraction and characterization of bioactive compounds from marine bacteria are challenging since they are produced in small amounts and often mixed forms [67]. As a result, metabolic engineering may need to integrate with natural product drug research for the large-scale manufacturing of the bioactive compounds in marine bacteria.

## 11. Concluding Remarks

**Figure 15:**
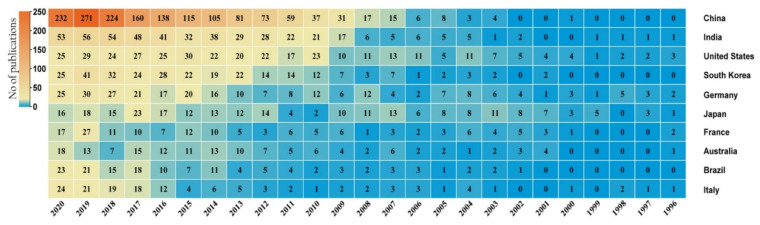
Statistical publication list from top ten countries engaged in studying marine bacteria for their antimicrobial activities. The data were retrieved from the PubMed database (https://pubmed.ncbi.nlm.nih.gov/, accessed on 25 July 2021) by providing the keywords “marine bacteria” and “antimicrobial activity” for the study period (1996–2020).

Finally, we attempted to examine the trends in scientific publications during the past twenty-five years (1996–2020) in countries engaged in identifying marine bacteria for their antimicrobial potentials. Scientific output in this area has increased rapidly, with the Asian continent leading the way. Moreover, the research organizations in China, India, the USA, South Korea, and Germany have become the top five significant prospectors in identifying antimicrobial-producing marine bacteria (Figure 15). It is clearly shown that the increasing focus of several countries on the identification of structurally unique and effective antimicrobial compounds from marine bacteria may indicate that a large number of new molecules may emerge as possible antimicrobial therapy options in the near future.

Overall, developing novel antimicrobial agents, and the alteration of existing drugs obtained from marine bacteria, will be crucial in fighting against microbial resistance. Considering all of the findings and technological advancements made over the past two and half decades, it is evident that marine bacteria will play a crucial role in the future development and trials of structurally unique and effective antimicrobial medicines.

## Figures and Tables

**Table 1:** Antimicrobial potential of marine bacteria derived from marine sediments/water.

S. No	Marine Source	Marine Bacteria	Secondary Metabolite(s)	Antimicrobial Activity	Ref
1	Sediment sample	*Nonomuraea* sp. MM565M-173N2	Sealutomicin A	Inhibited the growth of carbapenem-resistant Enterobacteriaceae	[155]
2	Sediment sample	*Streptomyces* sp.	-	Exhibited antifungal activity against *C. albicans*	[156]
3	Sediment sample	*Actinomycete* AMA50	Tetradecanoic acid, pentadecanoic acid, and n-hexadecanoic acid	Exhibited antifungal activity against *Talaromyces marneffei*	[157]
4	Sediment sample	*Streptomyces* sp. SY1965	Streptothiazolidine A	Exhibited antifungal activity against *C. albicans*	[158]
5	Sediment sample	*B. velezensis* SH-B74	Anteiso-C15 Ile2,7 surfactin, 1	Inhibited the appressoria formation of rice blast fungal pathogen *Magnaporthe oryzae*	[159]
6	Sediment sample	*Pseudomonas* sp.	Chitinase	Showed antifungal activity against *Verticillium dahlia* CICC 2534 and *Fusarium oxysporum f. sp. cucumerinum* CICC 2532	[160]
7	Sediment sample	*Brevibacillus antibioticus* sp. TGS2-1T	Different fatty acids and lipid compounds	Exhibited both antibacterial and antifungal activities	[161]
8	Sediment sample	*Nocardiopsis* sp. SCA21	4-bromophenol and bis (2-ethylhexyl) phthalate	Showed antibacterial activity against several Gram-positive and Gram-negative bacteria	[162]
9	Sediment sample	*Salinispora arenicola*	3-hydroxy-*N*-methyl-2-oxindole derivatives	Inhibited the growth of *E. faecalis*	[163]
10	Estuary soil sample	*S. felleus*	Polyketide compounds	Exhibited antibacterial activity against *Enterococcus* sp.	[164]
11	Sediment sample	*S. arenicola*	Salinaphthoquinones	Exhibited moderate antibacterial activity against *E. faecalis* and *S. aureus*	[165]
12	Sediment sample	*Streptomyces* sp.	Meroterpenoids	Showed intense antibacterial activity against *B. subtilis* and *S. aureus*	[166]
13	Sediment sample	*Streptomyces* sp. RKND004	Terrosamycins A and B	Exhibited strong antibacterial activity against Gram-positive bacteria	[167]
14	Sediment sample	*Streptomyces* strains	-	Exhibited antifungal activity against *C. albicans* and *A. niger*	[168]
15	Sediment sample	*Streptomyces* sp. 4054	Mycenolide A	Inhibited the growth of *B. subtilis*	[169]
16	Sediment sample	*S. xinghaiensis* SCSIO S15077	Tunicamycin derivatives	Showed both antibacterial and antifungal activities	[170]
17	Sediment sample	*Bacillus* and *Virgibacillus* strains	-	Exhibited antibacterial activity against *S. aureus* and *V. parahaemolyticus*	[171]
18	Sediment sample	Actinobacteria SJP4	[1,2,4]triazol-1-ylethanone	Exhibited antibacterial activity against both Gram-positive and Gram-negative bacteria	[172]
19	Sediment sample	*Streptomyces sp. Bacillus* sp. and *Micrococcus* sp.	-	Showed both antibacterial and antifungal activities	[173]
20	Sediment sample	*S. mutabilis* sp. MII	N-acetylborrelidin B	Exhibited both antibacterial and antifungal activities	[174]
21	Mangrove sediment sample	*Streptomyces*	-	Exhibited antibacterial activity against both Gram-positive and Gram-negative bacteria	[175]
22	Mangrove sediment sample	Actinobacteria	Extracellular enzymes	Exhibited both antibacterial and antifungal activities	[176]
23	Sediment sample	*Aneurinibacillus* sp. YR247	Peptidic compounds	Showed antifungal activity against *A. brasiliensis* NBRC945	[177]
24	Sediment sample	*Rheinheimera japonica* KMM 9513 T	Diketopiperazines	Inhibited the growth of *B. subtilis*, *S. aureus* and *E. faecium*	[178]
25	Sediment sample	*T. flavus* SP5	Different bioactive compounds	Inhibited growth of both bacterial and fungal pathogens	[179]
26	Mangrove sediment sample	*S. parvulus* DOSMB-D105	Different bioactive compounds	Exhibited both antibacterial and antifungal activities	[180]
27	Sediment sample	*R. japonica* sp.	Isoprenoid quinones	Exhibited strong antagonistic activity against both Gram-positive and Gram-negative bacteria	[181]
28	Sediment sample	*Streptomyces* sp. CMB-M0244	Mollemycin A	Exhibited antibacterial activity against both Gram-positive and Gram-negative bacteria	[182]
29	Sediment sample	*B. sonorensis* MT93	Sonorensin	Exhibited broad spectrum of antibacterial activity against different bacterial pathogens	[183]
30	Sediment sample	*Streptosporangium* sp. DSM 45942	Iodinin	Exhibited both antibacterial and antifungal activities	[184]
31	Sediment sample	*Streptomyces* sp. 12A35	Lobophorin I	Exhibited antibacterial activity against *S. aureus* and *B. subtilis*	[185]
32	Sediment sample	*Actinoalloteichus* sp. NPS702	Neomaclafungins	Showed strong antifungal activity against *Trichophyton mentagrophytes*	[186]
33	Sediment sample	*Pseudonocardia* sp. SCSIO 01299	Pseudonocardians A–C	Exhibited antibacterial activity against both Gram-positive and Gram-negative bacteria	[187]
34	Sediment sample	*Streptomyces* sp. NTK 937	Caboxamycin	Exhibited antibacterial activity against Gram-positive bacterial pathogens	[188]
35	Sediment sample	*B. laterosporus* Lh-1	-	Exhibited antibacterial activity against both Gram-positive and Gram-negative bacteria	[189]
36	Sediment sample	*Streptomyces* sp. BD21-2	Bonactin	Exhibited antibacterial activity against both Gram-positive and Gram-negative bacteria	[190]
37	Sediment sample	*S. koyangensis* SCSIO 5802	Neoabyssomicins	Exhibited antiviral activity against HSV and vesicular stomatitis virus	[191]
38	Sediment sample	*Streptomyces.* sp. #HK18	Xiamycin C	Exhibited strong antiviral activity against porcine epidemic diarrhea virus	[192]
39	Water sample	*P. putida*	9, 10-dihydrophenanthrene-2-carboxylic acid	Revealed strong antifungal activity against *C. albicans*	[193]
40	Water sample	*Bacillus, Arthrobacter*, and *Brevundimonas*	-	Exhibited antibacterial activity against both Gram-positive and Gram-negative bacteria	[194]
41	Water sample	*Pseudoalteromonas haloplanktis* TAC125	Methylamine	Inhibited the growth of *Burkholderia cepacia* complex	[195]
42	Estuarine water sample	Different strains of heterotrophic bacteria	-	Inhibited the growth of *S. aureus* and *E. coli*	[196]

**Table 2:** Antimicrobial potential of bacteria associated with different marine fauna.

S. No	Marine Fauna	Associated Marine Bacteria	Secondary Metabolite(s)	Antimicrobial Activity	Ref
1	Sponges (*H. panacea* and *Hymeniacidon perlevis*)	*Maribacter*, *Aquimarina, Vagococcus*, and *Denitrobaculum* bacterial isolates	-	Exhibited antibacterial activity against *S. aureus*	[228]
2	Sponges (*Agelas nakamurai* and *Aaptos suberitoides*)	Different *Bacillus* sp.	Macrolactin A and C14-surfactin	Inhibited the growth of *E. coli* and *M. luteus*	[229]
3	Sponge (*Suberea mollis*)	*Vibrio* sp. EA348	Fosfomycin and amifloxacin	Exhibited antibacterial activity against both Gram-positive and Gram-negative bacteria	[230]
4	Sponges (Demospongiae and Homoscleromorpha)	Genera *Vibrio* and *Bacillus*	-	Inhibited the growth of different multidrug-resistant bacterial pathogens	[231]
5	Sponge (*Arenosclera brasiliensis*)	Diversity of heterotrophic bacteria	-	Exhibited antibacterial activity against *B. subtilis*	[231]
6	Sponge (*Orina sagittaria*)	*Actinomycete* strain F-04	Volatile organic compounds	Inhibited growth of *S. aureus*	[232]
7	Coral (*Lophelia pertusa*)	*Streptomyces* sp. M-207	Lobophorin K	Exhibited antibacterial activity against *S. aureus*	[233]
8	Coral (*Platygyra* sp.)	*Pseudoalteromonas* sp.	Heat tolerant cell-free culture supernatant	Inhibited the growth of *B. cereus* and *S. aureus*	[234]
9	Coral (*Antipathes dichotoma*)	Three different bacterial phyla (*Actinobacteria, Alphaproteobacteria*, and *Firmicutes*)	-	Exhibited both antibacterial and antifungal activities	[235]
10	Mollusk (Oysters—*Crassostrea gigas*)	*Pseudoalteromonas* hCg-6 and hCg-42	Cyclolipopeptides	Inhibited the growth of Gram-negative human bacterial pathogens	[236]
11	Mollusk (*Batillaria zonalis*)	*S. sampsonii* SCSIO 054	Julichrome Monomers	Inhibited the growth of *S. simulans* and *S. aureus*	[237]
12	Mollusk (*Onchidium* sp.)	*S. olivaceus* SCSIO LO13	Borrelidins	Exhibited antibacterial activity against both Gram-positive and Gram-negative bacterial pathogens	[238]
13	Mollusk (*Kuphus polythalamius*)	*P. aeruginosa* 1682U.R.0a.27	Mindapyrroles A–C	Showed antibacterial activity against both Gram-positive and Gram-negative bacteria	[239]
14	Mollusk (*M. edulis*)	*Bacillus* sp. BC028	Bacicyclin	Inhibited the growth of clinical pathogens *S. aureus* and *E. faecalis*	[225]
15	Mollusk (*Lienardia totopotens*)	*Streptomyces* sp.	Lobophorins	Inhibited the growth of *M. tuberculosis* and *B. cepacia*	[240]
16	Mollusk (*Anadara broughtoni*)	*Saccharothrix espanaensis* An 113	Saccharothrixmicines A and B	Exhibited antifungal activity against *C. albicans*	[241]
17	Mollusk (*A. broughtoni*)	*B. pumilus* An 112	Cyclic depsipeptides	Exhibited broad spectrum antibacterial activity	[242]
18	Mollusk (*Pecten maximus*)	Bacterial strains CF-20 and C-148	dd-diketopiperazines	Exhibited antibacterial activity against *V. anguillarum*	[243]

**Table 3:** Antimicrobial potential of bacteria associated with different marine flora.

S. No	Marine Flora	Associated Marine Bacteria	Secondary Metabolite(s)	Antimicrobial Activity	Ref
1	Seaweed (*Gracilaria canaliculata*)	*Lysinibacillus odysseyi* KC149512	Lupenol, diazene, and furan	Inhibited the growth of Gram-negative bacterial pathogens	[275]
2	Seaweed (*Asparagopsis armata*)	*Shewanella* sp. ASP 26	-	Showed antibacterial activity against *S. aureus* and *B. subtilis*	[276]
3	Seaweeds (*U. lactuca*, *G. corticata*, and *Mastophora rosea*)	Phyla of Proteobacteria and Firmicutes	2-Pyrrolidinone, Phenol, 2, 4-bis (1, 1-dimethylethyl) and Furan derivatives	Exhibited antibacterial activity against clinical pathogens	[277]
4	Seaweed (*Ulva* sp.)	*S. althioticus* MSM3	Desertomycin G	Showed antibacterial activity against a broad range of Gram-positive bacterial pathogens	[278]
5	Seaweed (*Anthophycus longifolius*)	*B. subtilis* MTCC 10403	Aryl-crowned polyketides	Exhibited antibacterial activity against different Gram-negative bacterial pathogens	[279]
6	Seaweed (*Pelvetia canaliculata*)	*K. marina* CMG S2	Kocumarin	Inhibited the growth of both bacterial and fungal pathogens	[280]
7	Seaweed (*P. gymnospora*)	*B. amyloliquefaciens*	Polyketides	Exhibited antibacterial activity against *V. vulnificus* and *V. parahaemolyticus*	[281]
8	Seaweed (*A. longifolius*)	*B. subtilis* MTCC 10403	Polyketide furanoterpenoids	Exhibited antibacterial activity against perceptive food pathogens	[282]
9	Seaweed (*Sargassum myriocystum*)	*B. subtilis* MTCC 10407	O-heterocyclic polyketide derivatives	Showed potent antibacterial activity against *V. parahaemolyticus*, *Aeromonas hydrophila*, and *V. vulnificus*	[283]
10	Seaweed (*Laurenciae papillosa*)	*B. amyloliquefaciens*	Polyketides	Inhibited the growth of food-borne pathogens	[284]
11	Seaweed (Rhodophyceae and Phaeophyceae)	Phyla of Firmicutes and Proteobacteria	Polyketides	Prevented the growth of fouling bacteria	[285]
12	Seaweed (*P. pavonica*)	*B. pumilus* P8	-	Exhibited both antibacterial and antifungal activities	[286]
13	Seaweed (*A. longifolius*)	*B. subtilis* MTCC 10403	7-O-methyl-5′-hydroxy-3′-heptenoate-macrolactin	Exhibited antibacterial activity against human opportunistic clinical pathogens	[287]
14	Seaweed (*Fucus serratus*)	*B. licheniformis*	YbdN protein	Inhibited the growth of *S. aureus* and *Listeria monocytogenes*	[288]
15	Seagrasses (Cymodocea sp., *Enhalus acoroides*, *Syringodium* sp., and *Thalassia hemprichii*)	*B. flexus* EED 15 and *S. lienomycini* EED 16	-	Inhibited the growth of *E. coli* and *S. aureus*	[289]
16	Seagrasses (*Cymodocea serrulata* and *Syringodium isoetifolium*)	Different endo and epiphytic bacteria	-	Inhibited the growth of different human bacterial pathogens	[290]
17	Mangrove (*Rhizophora mucronata*)	*Rhodococcus* sp.	Sterol-glycosides	Exhibited antibacterial activity against aquatic bacterial pathogens	[291]
18	Mangroves (Seven different)	Belongs to the phylum Gammaproteobacteria	-	Showed antifungal activity against fungal pathogens	[292]
19	Mangrove (*Avicennia marina*)	*V. parahaemolyticus*	Vibriocin	Used in the management of controlling the vibrio infections	[293]
